# A case of pancreatic PEComa with prominent inflammatory cell infiltration: the inflammatory subtype is a distinct histologic group of PEComa

**DOI:** 10.1186/s13000-024-01485-2

**Published:** 2024-04-15

**Authors:** Hikaru Tsukita, Kei Koyama, Takahiro Ishinari, Ayana Takahashi, Ken Miyabe, Michinobu Umakoshi, Makoto Yoshida, Yukitsugu Kudo-Asabe, Akiko Nishida, Naohiko Otsuka, Ouki Yasui, Ikuma Kato, Noriyoshi Fukushima, Akiteru Goto

**Affiliations:** 1 Department of Cellular and Organ Pathology, Akita University Graduate School of Medicine, 1- 1-1 Hondo, Akita-shi, Akita, 010-8543 Japan; 2 Department of Pathology, Nihonkai General Hospital, Sakata, Japan; 3 Department of Pathology, Akita City Hospital, Akita, Japan; 4 Department of Surgery, Noshiro Yamamoto Medical Association Hospital, Noshiro, Japan; 5 Department of Molecular Pathology, Yokohama City University Graduate School of Medicine, Yokohama, Japan; 6 Department of Pathology, Jichi Medical University, Shimotsuke, Japan

**Keywords:** PEComa, Inflammatory subtype, Pancreatic tumor, αSMA, Melan A, HMB45, Case report

## Abstract

**Background:**

PEComa is a mesenchymal tumor that can occur in various organs including the uterus and soft tissues. PEComas are composed of perivascular epithelioid cells, and angiomyolipoma (AML), clear cell sugar tumor (CCST), and lymphangiomyomatosis (LAM) are considered lesions of the same lineage as tumors of the PEComa family. Histologically, a common PEComa shows solid or sheet-like proliferation of epithelioid cells. This is accompanied by an increase in the number of dilated blood vessels. Here, we report a case of pancreatic PEComa with marked inflammatory cell infiltration.

**Case presentation:**

A 74-year-old male patient underwent an appendectomy for acute appendicitis. Postoperative computed tomography and magnetic resonance imaging revealed a 30 × 25 mm non-contrast-enhanced circular lesion in the tail of the pancreas. The imaging findings were consistent with a malignant tumor, and distal pancreatectomy was performed. Histologically, most area of the lesion was infiltrated with inflammatory cells. A few epithelioid cells with large, round nuclei, distinct nucleoli, and eosinophilic granular cytoplasm were observed. Spindle-shaped tumor cells were observed. Delicate and dilated blood vessels were observed around the tumor cells. Immunohistochemically, the atypical cells were positive for αSMA, Melan A, HMB-45, and TFE3. The cytological characteristics of the tumor cells and the results of immunohistochemical staining led to a diagnosis of pancreatic PEComa.

**Conclusions:**

A histological variant known as the inflammatory subtype has been defined for hepatic AML. A small number of tumor cells present with marked inflammatory cell infiltration, accounting for more than half of the lesions, and an inflammatory myofibroblastic tumor-like appearance. To our knowledge, this is the first report of pancreatic PEComa with severe inflammation. PEComa is also a generic term for tumors derived from perivascular epithelioid cells, such as AML, CCST, and LAM. Thus, this case is considered an inflammatory subtype of PEComa. It has a distinctive morphology that is not typical of PEComa. This histological phenotype should be widely recognized.

## Background

A PEComa is a mesenchymal neoplasm composed of perivascular epithelioid cells that occurs in the uterus, soft tissues, and other organs [1]. It is also a general term used for tumors of perivascular epithelioid cells. Angiomyolipoma (AML) of the liver and kidney, clear cell sugar tumor (CCST), and lymphangiomyomatosis (LAM) of the lung are all considered lesions of the same lineage [2–5]. Histologically, epithelioid cells with eosinophilic granular cytoplasm formed nested, trabecular, and sheet-like structures. Some cells showed a spindle cell morphology. Dilated capillaries were observed around the tumor and various proportions of adipocytes were present. A few cases of hepatic AML with prominent inflammatory cell infiltration have been reported and are defined as inflammatory subtypes [6, 7]. PEComa of the pancreas is relatively rare, with approximately 30 cases reported to date; however, none have marked inflammatory cell infiltration. Herein, we report a case of pancreatic PEComa corresponding to an inflammatory subtype.

## Case presentation

**Fig. 1:**
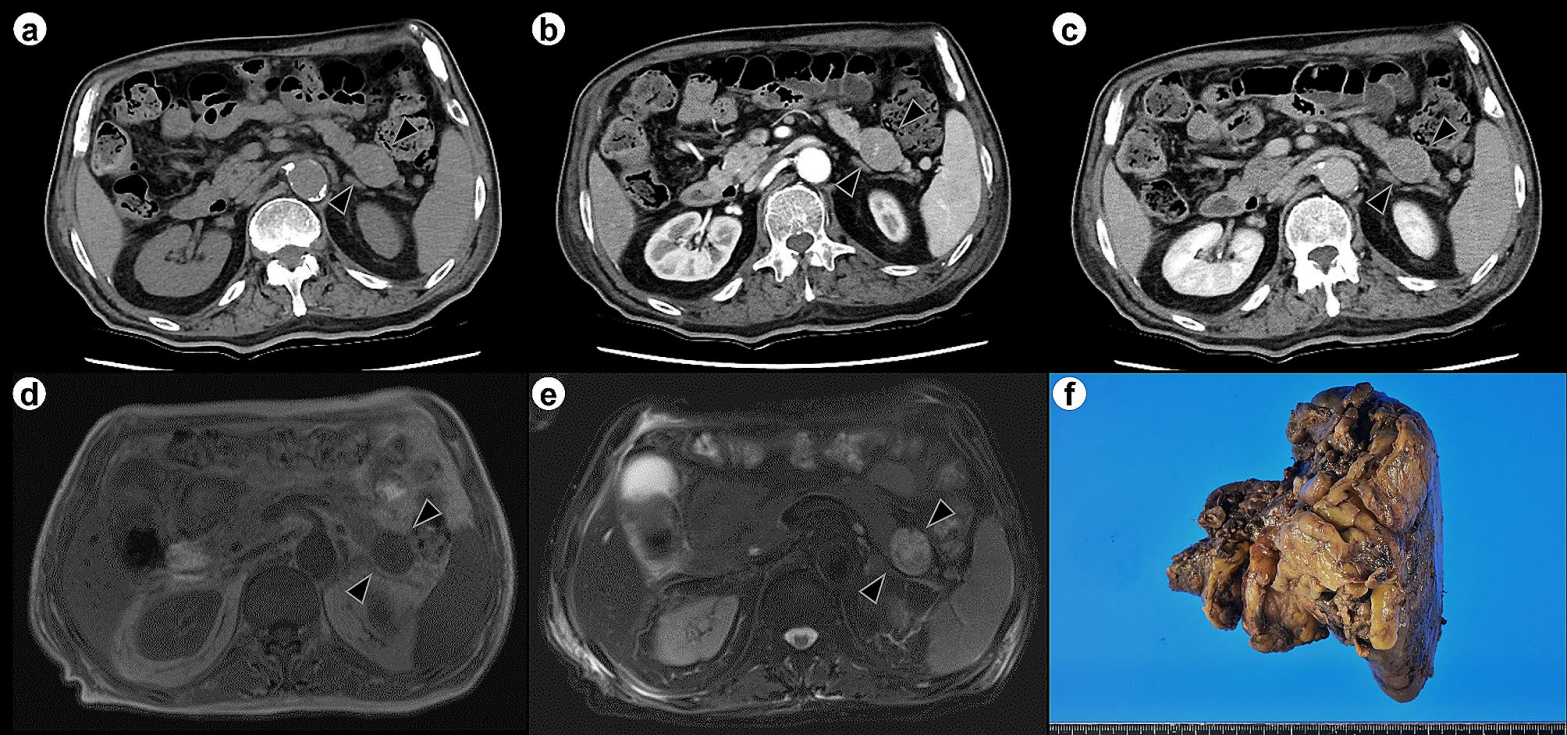
Abdominal CT and MRI of the pancreatic mass and gross view of the resected specimen. Arrowheads on CT and MRI show the tumor. Abdominal non-enhanced CT shows a solid lesion in the pancreatic tail (**A**). In the arterial phase of contrast-enhanced abdominal CT, the pancreatic mass does not show contrast enhancement (**B**). In the excretory phase of contrast-enhanced abdominal CT, enhancement is observed at the margins of the pancreatic mass (**C**). The mass demonstrates low signal intensity on T1-weighted images (**D**) and high signal intensity on T2-weighted images (**E**). Gross view of the resected specimen. The solid tumor is detected in the pancreatic body. The spleen is enlarged (**F**)

A 74-year-old male patient presented with abdominal pain for several days and was urgently transported to the hospital. The patient did not have any features of tuberous sclerosis complex. Abdominal computed tomography (CT) imaging revealed acute perforated appendicitis. The patient was hospitalized and underwent appendectomy. Histologically, the appendectomy specimen revealed phlegmonous acute appendicitis. Inflammation partially reached the serosa, indicating focal peritonitis. Post-operative CT and magnetic resonance imaging (MRI) showed a 30 × 25 mm circular lesion without a contrast effect in the pancreatic tail, incidentally (Fig. 1). The tumor was confined to the pancreas and had no extrapancreatic extension. Imaging revealed an enlarged spleen, without any signs of neoplastic lesions. A blood test showed that the levels of CEA and CA19-9 (tumor markers) were 1.6 ng/dL (< 5.0ng/dL) and 152.7U/mL (< 37.0U/mL), respectively. The imaging procedure findings were consistent with a malignant tumor of the pancreas, and distal pancreatectomy was performed. Thirteen months after the surgery, no local recurrence or distant metastasis was observed.

Macroscopically, there was a 27 × 25 mm mass in the tail of the pancreas. The section showed a well-circumscribed solid yellowish-white tumor with black spots. The tumor did not invade the pancreas and appeared to have been removed. The spleen was 120 × 110 × 50 mm in size and significantly enlarged. No tumor involvement was observed in the spleen.

**Fig. 2:**
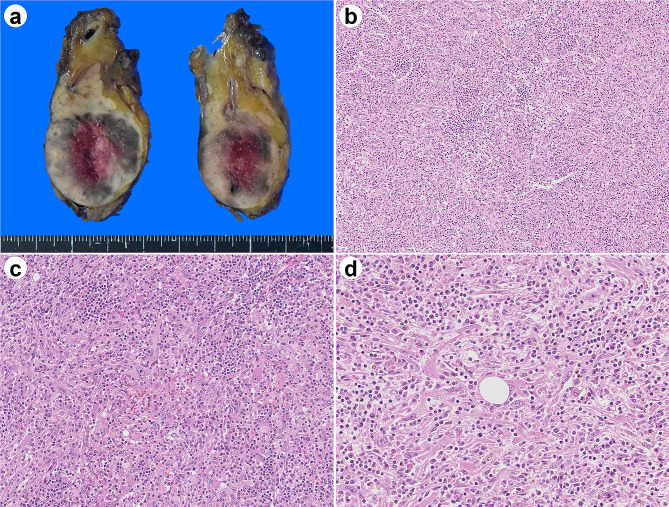
Pathological findings of the pancreatic tumor. The cut surface of the surgical specimen shows a solid yellowish-white tumor. The mass is well demarcated and localized to the pancreas (**A**). Marked inflammatory cell infiltration is also observed. At low magnification, the tumor cells are obscured by inflammatory cells. Slit-like dilated vessels are scattered (**B**). The majority of the lesion is composed of inflammatory cells. A few epithelioid cells with large round nuclei, distinct nucleoli, and eosinophilic granular cytoplasm are observed. Atypical spindle cells are also observed (**C**). Some tumor cells exhibit capillary-like structures (**D**)

Microscopically, the lesion was mainly composed of inflammatory cellular infiltrates. They are predominantly lymphocytes and plasma cells, with a mixture of neutrophils and macrophages, including a small number of eosinophils. Slightly increased and dilated capillaries were also observed. Brownish granular deposits were observed in macrophages and interstitium. A small number of atypical cells with large round nuclei, distinct nucleoli, and eosinophilic granular cytoplasm were observed in a highly scattered pattern. These atypical cells showed epithelioid or spindle-shaped morphology. Some formed luminal structures resembling capillaries (Fig. 2). There was no adipose tissue or thick-walled sclerosed vessels, as is typically observed in AMLs. The margins of the lesion consisted of inflammatory cells and small amounts of scattered tumor cells, and the border with the surrounding pancreatic tissue was relatively clear. There was no extrapancreatic spillover of inflammation or tumor cells. There was no mitosis per 50 high-power fields (HPFs) and there was no lymphovascular invasion. No tumor necrosis was observed. Non-contiguous lesions with similar clusters of inflammatory cells were found on the pancreatic tail of the main lesion. Fibrosis consisting of collagen fibers was observed in the background interstitium; however, storiform fibrosis was not observed. However, no atypical cells were observed. The spleen was congested, but there was no evidence of tumor spread or metastasis.

**Fig. 3:**
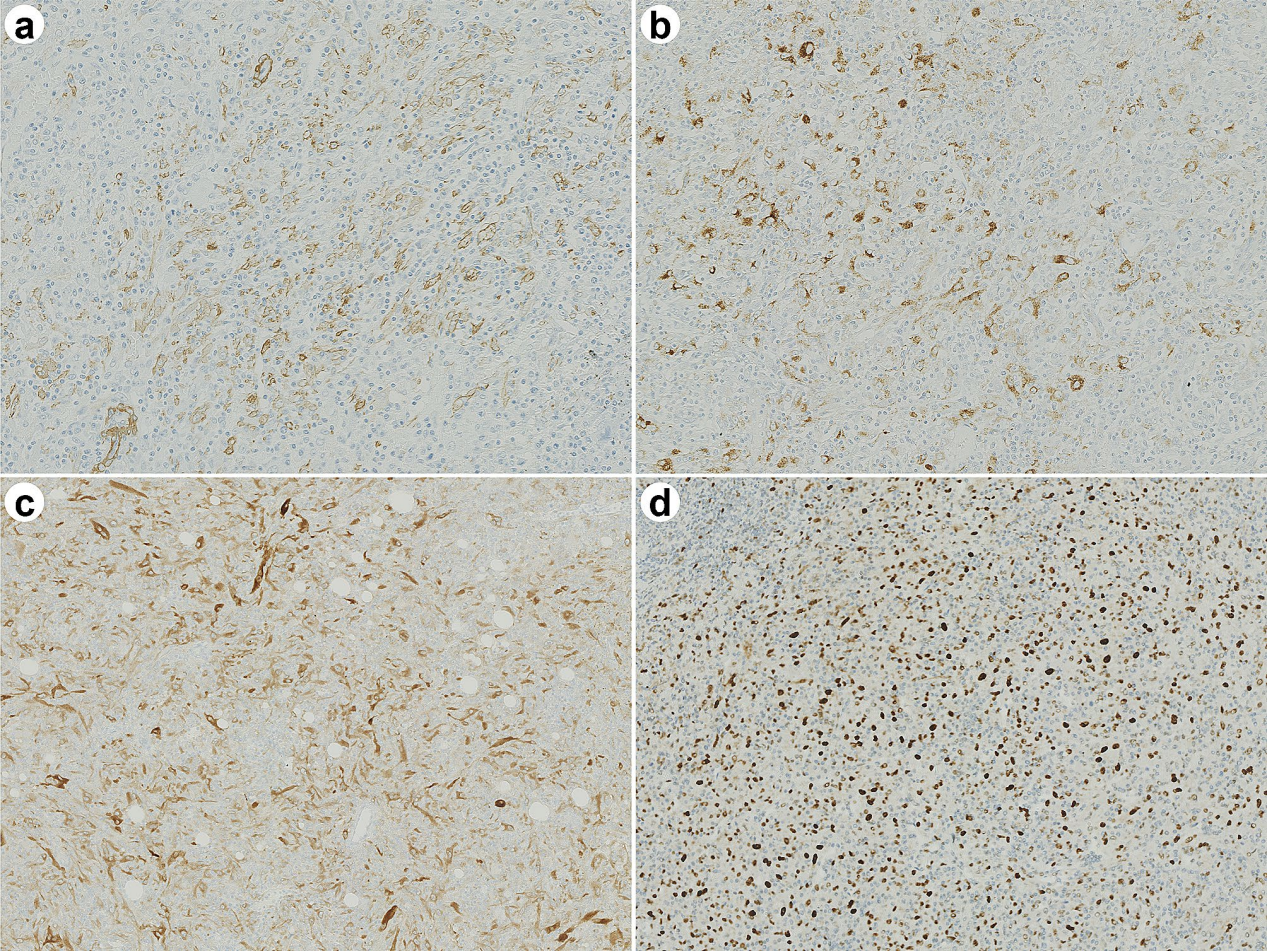
Immunohistochemical staining of tumor cells. α-SMA is detected in the plasma membranes of some tumor cells. Delicate blood vessels within the lesion are highlighted (**A**). HMB-45 is granular-positive in the cytoplasm (**B**). Melan A staining is positive in the cytoplasm (**C**). TFE3 is diffusely positive in the nucleus (**D**)

Immunohistochemically, atypical cells were positive for vimentin, αSMA (focal and weak), Melan A, HMB-45, TFE3, Cathepsin K, and negative for CK AE1/AE3, S-100, Desmin, CD21, CD23, and ALK. EBER-ISH results were negative (Fig. 3; Table 1). The expressions of MLH1, PMS2, MSH2, and MSH6 were retained. The plasma cells in the lesions expressed IgG and IgG4. At the hot spots, the number of IgG4 + plasma cells was 22–28/HPF, with an IgG4/IgG ratio of 17.5–28.8%.

**Table 1:** Summary of immunohistochemistry

Antibody	Result		Antibody	Result
Vimentin	Positive		CD23	Negative
αSMA	Positive (focal and weak)		CD30	Negative
Melan A	Positive		CD35	Negative
HMB-45	Positive		CD45RO	Negative
TFE3	Positive		CD68	Negative
Cathepsin K	Positive		IgG	Negative
Cytokeratin (AE1/AE3)	Negative		IgG4	Negative
Desmin	Negative		MLH1	Retained
S-100	Negative		PMS2	Retained
Podoplanin (D2-40)	Negative		MSH2	Retained
ALK	Negative		MSH6	Retained
CD1a	Negative		EBER-ISH	Negative
CD21	Negative			

IgG4 + plasma cells: 22–28/HPF, IgG4/IgG ratio 17.5–28.8%

## Discussion and conclusions

PEComa is a group of tumors originating from perivascular epithelioid cells, which exist perivascularly and have multi-differentiation potential [1, 8]. Tumors such as AML, pulmonary CCST, and LAM are included and have been called by different names depending on their morphology and the organ in which they arise [2–5]. The disease concept of the PEComa family began in 1992 when Bonetti et al. described tumor cells common to these tumors as being of perivascular epithelioid cell origin [9]. In the pancreas, it is commonly referred to as PEComa and is a relatively rare type of primary pancreatic tumor [10]. In a study by Kim et al., 1 of the 7129 pancreatic tumors was AML [11].

Pancreatic PEComa is characterized by sheets or nests of epithelioid cells with clear or eosinophilic granular cytoplasm surrounded by a delicate capillary vascular network. The majority of cases were dominated by epithelioid cells, with a spindle cell component observed in a minority of cases [10]. In general, a PEComa has medullary morphology. In this case, however, a small number of epithelioid or spindle tumor cells were observed hidden in a cluster of inflammatory cells. The tumor cells had eosinophilic granular cytoplasm and large round nuclei, with strong nuclear atypia. Based on the morphological findings of spindle-shaped or macrophage-like round atypical cells with high inflammatory cell infiltration, inflammatory myofibroblastic tumor and follicular dendritic cell sarcoma were considered as differential diagnoses. The growth of delicate blood vessels and thick-walled blood vessels was conspicuous; therefore, we suspected vascular tumors. The tumor cells were immunohistochemically negative for ALK and follicular dendritic cell markers such as CD21, CD23, and CD35, and positive for αSMA, Melan A, and HMB-45. The cytological characteristics of the tumor cells, surrounding vascular growth, and immunohistochemical positivity for smooth muscle and melanocytic markers led to the diagnosis of PEComa. Folpe et al. proposed a histopathological grading scheme for soft tissue and uterine PEComa based on the histological features of tumor size > 50 mm, high nuclear grade, mitotic rate > 1/50 HPFs, necrosis, vascular invasion, and infiltrative growth [4]. This case was considered to have “uncertain malignant potential” because only a high nuclear grade was observed, and no other histological features of malignant PEComa was observed.

WHO tumor classifications of digestive systems describes the “inflammatory subtype of angiomyolipoma,” as chronic inflammatory cell infiltration that constitutes 50% or more of the tumor and has been reported mainly in the liver [12]. It is a very rare subtype, and approximately 15 cases have been reported since the report by Kojima in 2004 [6, 13]. Macroscopically, the lesion was well-circumscribed with a pushing border. The majority of lesions showed inflammatory cell infiltration, and some epithelioid tumor cells and spindle cells were present. Delicate dilated vessels were present, similar to those observed in typical AML. Cavernous-like sinusoidal vessels were also observed [14]. The presence of mature adipocytes varied according to previous reports [15, 16]. As is common in AML, tumor cells are positive for smooth muscle and melanocytic markers. It has been suggested that TFE3 may be useful for the diagnosis [17].

An association between hepatic angiomyolipoma and IgG4-related disease (IgG4-RD) was suggested in the previous report [14]. In this case, foci of inflammatory cells were found not only in the tumor area but also in the pancreatic parenchyma at some distance from the tumor. Blood IgG and IgG4 levels were not measured before the surgery. No significant increase in the number of IgG4-positive plasma cells or IgG4/IgG ratio was observed in either lesion, and this case did not meet the diagnostic criteria for IgG4-RD [18, 19].

Histologically, this case is characterized by an intense inflammatory cell infiltrate; however, the results of immunohistochemical staining for mismatch repair proteins suggest that their functional defects are not the cause, as in colonic medullary carcinoma. As with the inflammatory subtype of AML, the mechanism underlying the prominence of inflammatory cell infiltration is unknown, and further studies are warranted.

Here, we present a case of pancreatic PEComa. There are no reported cases of this morphology in the group of diseases called PEComa, which includes pancreatic origin, as far as we have been able to investigate. Its morphology is similar to that of the inflammatory AML subtype. It is very strange that the inflammatory subtype is defined only in AML, as AML is one of the subtypes of the PEComa family, along with CCST and LAM. We believe that this case appropriately describes the inflammatory subtype of pancreatic PEComa. In general, PEComa is considered a benign lesion; however, in some cases, it recurs. This histological subtype is very rare, and its clinical features, including prognosis, remain unclear. Although it has been determined to have an uncertain malignant potential according to the criteria of Folpe et al., we do not know if we can estimate the biological potential of this histological type [4]. In this case, the association with IgG4-RD was unknown. It is hoped that further studies will provide a better understanding of the clinical characteristics of PEComas and their causes.

## Data Availability

No datasets were generated or analysed during the current study.

## Abbreviations

AML: Angiomyolipoma
CCST: clear cell sugar tumor
LAM: lymphangiomyomatosis
CT: computed tomography
MRI: Magnetic resonance imaging
HPF: high-power field
IgG4-RD: IgG4-related disease
